# Supplementary material for: New Insights into the Mechanism of Spatiotemporal Scent Accumulation in Orchid Flowers
Source: Plants (Basel). 2023 Jan 9;12(2):304. doi: 10.3390/plants12020304 (PMC9866394; doi:10.3390/plants12020304)
Supplement: Supplementary file 1 [file plants-12-00304-s001.zip › plants-2046229-supplementary.pdf]

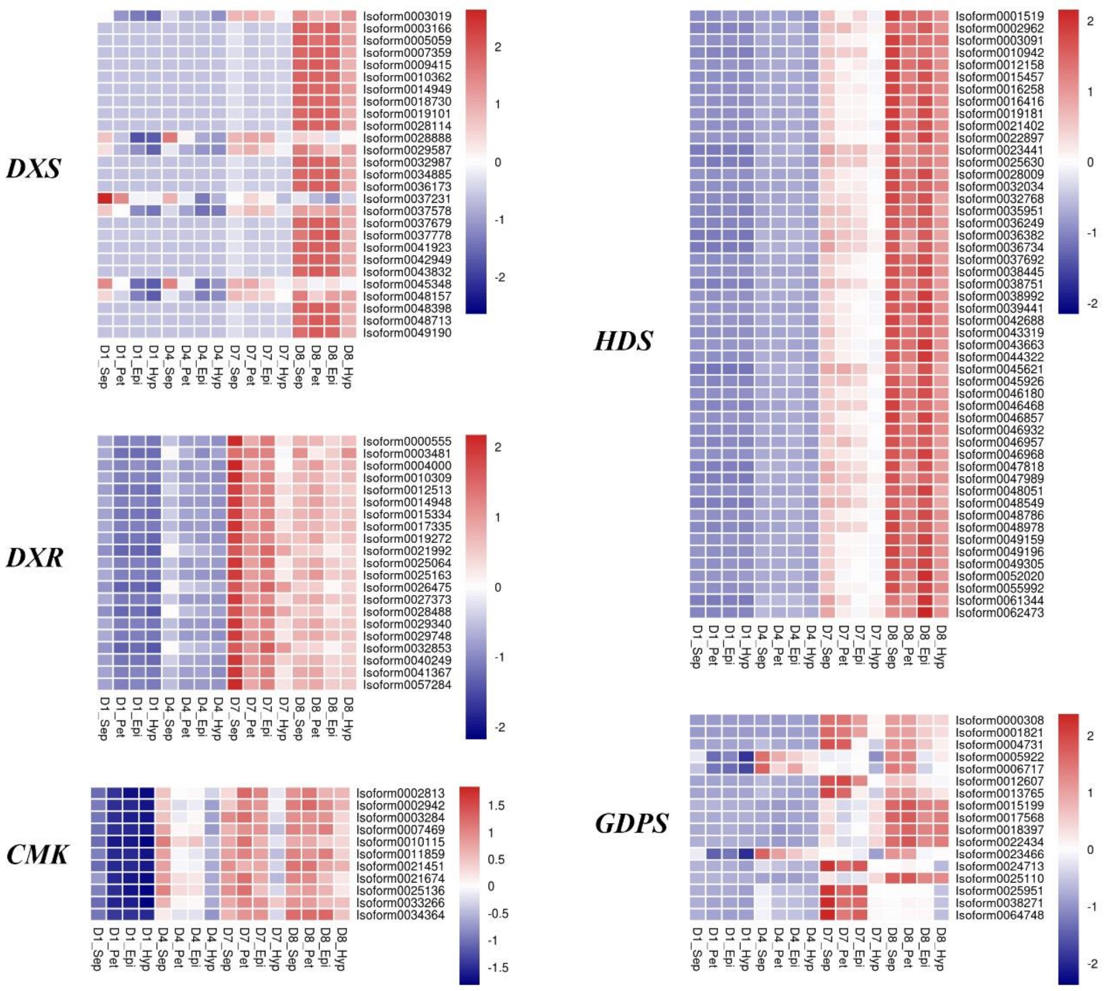

Supplementary Figure 1 The spatiotemporal expression profiles of partial MEP pathway structural genes. According to a previous study, KOVA flower development is classified into eight stages, including D1 (i.e., bub length < 2 cm), D4 (i.e., bub length 4–5 cm), D7 (i.e., two days after flowering), and D8 (i.e., ten days after flowering) (Li et al., 2020). A heatmap analysis of the expression levels of partial MEP pathway structural genes in the sepals, petals, epichile, and hypochile from D1 (developmental stage 1) to D8 is displayed. The transcriptome data were provided by a previous study (Li et al., 2020) and reanalyzed in this study. The data are the mean ± SD from three biological replicates. Abbreviations for enzymes are as follows: 1-deoxy-d-xylulose 5-phosphate synthase (DXS), 1-deoxy-d-xylulose-5-phosphate reductoisomerase (DXR), 4-(cytidine 5'-diphospho)-2-C-methyl-D-erythritol kinase (CMK), 4-hydroxy-3-methylbut-2-enyl diphosphate synthase (HDS), geranyl diphosphate synthase (GDPS).

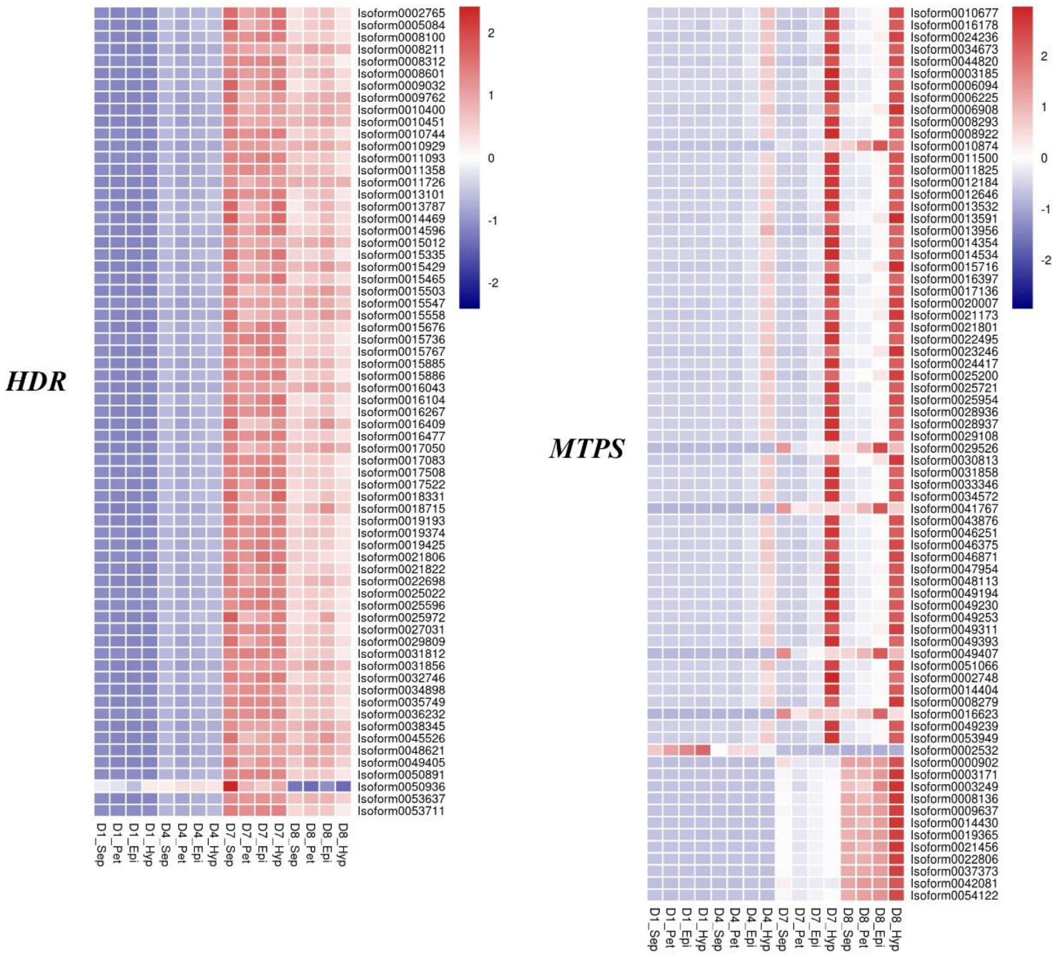

Supplementary Figure 2 The spatiotemporal expression profiles of partial MEP pathway structural genes. According to a previous study, KOVA flower development is classified into eight stages, including D1 (i.e., bub length < 2 cm), D4 (i.e., bub length 4–5 cm), D7 (i.e., two days after flowering), and D8 (i.e., ten days after flowering) (Li et al., 2020). A heatmap analysis of the expression levels of partial MEP pathway structural genes in the sepals, petals, epichile, and hypochile from D1 (developmental stage 1) to D8 is displayed. The transcriptome data were provided by a previous study (Li et al., 2020) and reanalyzed in this study. The data are the mean ± SD from three biological replicates. Abbreviations for enzymes are as follows: 1-hydroxy-2-methyl-2-(E)-butenyl 4-diphosphate reductase (HDR), monoterpene synthase (MTPS).
